# The Role of Laparoscopy in the Management of a Diagnostic Dilemma: Jejunal Ectopic Pancreas Developing into Jejunojejunal Intussusception

**DOI:** 10.1155/2017/8452947

**Published:** 2017-07-27

**Authors:** Alessio Giordano, Giovanni Alemanno, Carlo Bergamini, Paolo Prosperi, Alessandro Bruscino, Andrea Valeri

**Affiliations:** General, Emergency and Minimally Invasive Surgery Unit, Careggi University Hospital, Florence, Italy

## Abstract

Ectopic pancreas (EP) is a rare congenital anomaly defined as the presence of pancreatic tissue in topographic anomaly. It is usually silent but it may become clinically evident when complicated by acute conditions. The development of laparoscopic surgery has changed the way to manage such conditions, especially in the setting of emergency surgery, thanks to its diagnostic and therapeutic role with excellent results. We decided to perform an emergency diagnostic exploratory laparoscopy in a 29-year-old man with an acute abdomen and nonspecific radiological images for intestinal occlusion. A jejunojejunal intussusception was found, caused by a mass. We decided to carry out minilaparotomy to perform a resection of the affected jejunum. Histological examination confirmed the presence of a jejunal ectopic pancreas. Adult intussusception caused by EP represents 5% of all cases of intussusception. As CT scan, especially when performed in emergency setting for small bowel obstruction diagnosis, can usually demonstrate nondiagnostic findings suggestive of intussusception of unknown origin, laparoscopic exploration could help surgeons in order to perform a resolute diagnosis and treat the pathology.

## 1. Introduction

Ectopic pancreas (EP) is defined as an anatomical abnormality in which pancreatic tissue has grown outside its normal location with no anatomical, neural, or vascular connection to the normal pancreas [1]. The EP is a relatively uncommon congenital abnormality with a range of incidence between 0.55% and 13.7% in autopsy series. EP has been found predominantly in the fourth-sixth decade of life [2] and it has rarely been reported in pediatric cases [3]. The usual locations of EP are the stomach in 25–38% cases, the duodenum in 17–36% of cases, and the jejunum in 15–22% of cases [4]. It is usually silent; in fact in the majority of cases it has been found incidentally at laparotomy or laparoscopy performed for other abdominal pathologies, but it may become clinically evident when complicated by acute conditions such as inflammation (pancreatitis), acute/subacute abdominal pain, bleeding, obstruction, or malignant transformation [5]. Malignant transformation can occur in the EP as well as in the tissue of a normally located pancreas. However, the incidence of tumor in EP is less than in normal pancreas tissue [6].

The development of laparoscopic surgery has changed the way to manage such conditions, especially in some setting of emergency surgery. In particular, laparoscopy may have three different roles: to validate the pathophysiologic diagnosis (diagnostic laparoscopy), to facilitate and guide a subsequent laparotomy (laparoscopic assisted open approach) or, finally, to entirely treat the disease (fully laparoscopic approach). Actually, in such condition, the results of laparoscopy approach are reported to be excellent in terms of reduction of hospital stay, incidence of surgical site infection, postoperative pain, and recovery of bowel function [7, 8].

While EP and intussusception are not unusual conditions, the intussusception caused by EP is rare [9]. We report a case of jejunal intussusception caused by EP in young man and describe the role of laparoscopy in emergency setting in order to guide the diagnosis with a successful surgical management.

## 2. Case Report

A 29-year-old man (BMI: 32 kg/m2) was admitted to our Emergency Department for nausea and intermittent abdominal pain. The pain had increased for the last 2 days in severity and was associated with episodes of emesis. The patient had a story of undetermined colitis, treated with medical therapy, actually in phase of remission.

**Figure 1:**
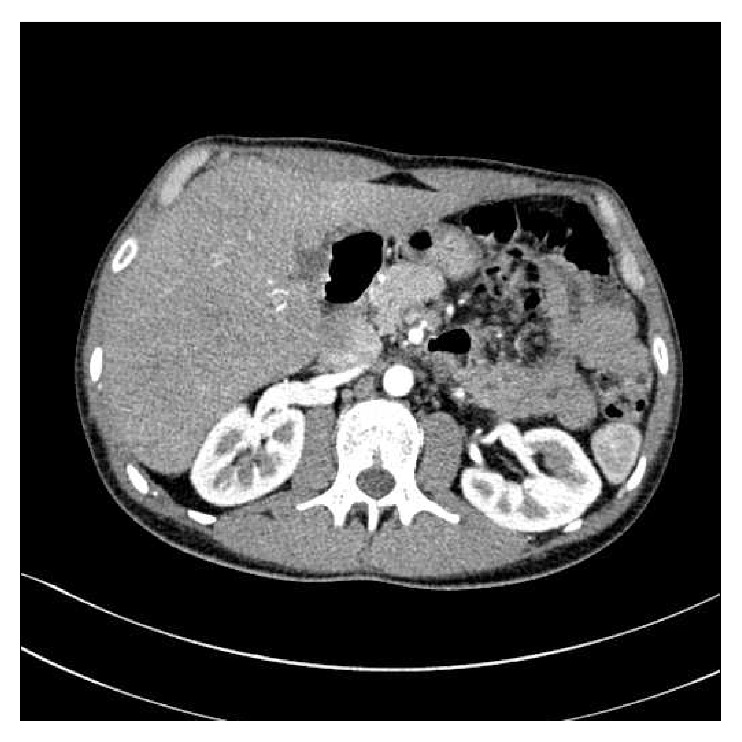
CT scan shows an aspecific thickening of jejunum with partial contrastographic enhancement near the left colon, with a modest dilatation of the lumen of small bowel upstream of the injury.

At clinical examination, the abdomen appeared flat, with a generalized tenderness, painful to palpation, in particular in the left upper quadrant. Lab tests showed a white blood cell count of 15,400/mm3. Chest and abdomen X-Rays were normal and abdominal ultrasound was normal too. On the contrary, the CT scan with intravenous contrast medium revealed an aspecific thickening of a tract of small bowel with partial contrastographic enhancement near the left colon, with a modest dilatation of the lumen of small bowel upstream of the injury, as reported in Figure 1.

Therefore, in order to perform a resolute diagnosis, in presence of an inconclusive and nondiagnostic CT scan, we decided to perform an emergency exploratory diagnostic laparoscopy. In the operating theater, after the first laparoscopic entry in the abdomen according to the open Hasson technique, an exploratory laparoscopy was performed. All the abdominal cavity was explored and all the bowel was inspected: at 35 cm from the Ligament of Treitz, a jejunojejunal intussusception was found. We decided to reduce the jejunal intussusception in order to define the pathologic cause, suspecting the presence of a mass. In fact, a mass of 4 cm in diameter was discovered on the intestinal serosal surface of the antimesenteric side and the mesentery presented multiple lymph nodes. The wall of the bowel was normal and no signs of ischemia were present.

In order to treat the cause of jejunal intussusception and to perform a histological diagnosis too, we decided to carry out an 8 cm laparotomy in the left upper abdominal quadrant and perform a resection of the affected jejunum (including the mass) and subsequently a jejunojejunal anastomosis.

The postoperative course was uneventful and the patient was discharged on the 5th postoperative day. Histological examination showed that the mass was a jejunal ectopic pancreas and that dissected lymph nodes simply share a lymphoid hyperplasia. According to the Heinrich classification system, this was a type 2 EP containing acini and ducts but no islets.

## 3. Discussion

The first case of EP was reported by Schultz in 1729 and Klob provided its histological confirmation in 1859 [5]. EP is defined as the presence of pancreatic tissue lacking anatomical and vascular continuity with the pancreas [1] and occurs in 0.5–13.7% of patients (these data are based on both autopsy and surgical series) [2]. In adults, EP had been found in all age groups, predominantly in the fourth-sixth decade of life, and its incidence is higher in males [2], while in children, there are very few reports and the female gender seems to prevail [3–5].

Usually EP is localized in the stomach (25–38%), duodenum (17–36%), and jejunum (15–22%), but it has been reported very rarely in other locations such as ileum, Meckel's diverticulum, colon, gallbladder, umbilicus, fallopian tube, mediastinum, spleen, and liver [4].

The etiology of EP is actually unknown even if multiple theories had been implicated on the embryological origin of this rare condition. One theory supposes the persistence of a duodenal evagination involved in the normal development of pancreas, so the remnant part might migrate with the developing of the gastrointestinal tract accounting for its various locations, while another theory suggests the existence of a pancreatic metaplasia of the endodermal tissue [10].

EP usually presents in the form of small yellowish nodules, ranging from 1 mm to 5 cm, covered by intact mucosa with rudimentary pancreatic duct and is frequently classified according to the Heinrich classification system (Table 1).

Most patients with EP are asymptomatic and diagnosis is usually performed during radiology or digestive endoscopy tract examination or surgical exploration for other diseases. When symptomatic, about 30% of cases present with abdominal pain due to pancreatitis, nausea, vomiting, anorexia, and bleeding. Conversely, intestinal obstruction with intussusception is rare [9]. Adult intussusception caused by EP represents 5% of all cases of intussusception and accounts for only 1–5% of intestinal obstruction in adults [4].

The diagnosis of EP still remains challenging and the preoperative imaging studies (ultrasonography, endoscopy, and CT scan) are not very specific [5].

Contrast-enhanced computed tomography, especially when performed in emergency setting for small bowel obstruction diagnosis, can usually demonstrate nondiagnostic findings such as exophytic bowel wall lesions or mural wall thickening suggestive of intussusception of unknown origin, like what happened in the case of our patient [31]. Therefore, the role of laparoscopic exploration to perform the diagnosis and treat the pathology seems to be very relevant. Furthermore, after the confirmation of the diagnostic suspect of intussusception, in our case, the subsequent laparotomic operation was surely facilitated by laparoscopy, which in fact allowed us to avoid a total xifopubic median incision and a long manual manipulation time until intussuscepted intestinal tracts were identified.

In Table 2, we report a literature review of all studies performed during the last 10 years about the EP and its treatment.

As the use of diagnostic and therapeutic laparoscopy improves postoperative outcome of the patient if compared to patients submitted directly to laparotomy, also in our case the patient was discharged on the fifth postoperative day with a rapid recovery of bowel function. Besides the reduction of postoperative hospital stay, laparoscopy generally is associated with significant reduction of postoperative analgesia, incidence of surgical site infections, postoperative cardiac and respiratory complications including pneumonia, and significant reduction of postoperative mortality rates [31–33].

## 4. Conclusion

EP is a rare congenital lesion often incidentally diagnosed on pathological examination and should be considered in the differential diagnosis of intestinal mass lesions, especially in case of acute complication such as intussusception. The treatment of intussusception in adults consists of resection of the intussuscepted mass. The laparoscopy has been demonstrated to be a safe and feasible alternative to directly open surgery since, along with its usual advantages, its diagnostic role is making it an attractive option, especially in emergency setting, in hemodynamically stable patient with nonconclusive imaging.

## Figures and Tables

**Table 1:** Heinrich classification system.

Type 1	EP containing acini, islets, and ducts
Type 2	EP containing acini and ducts, with no islets
Type 3	EP containing duct alone
Type 4	EP containing islets alone

**Table 2:** Review of literature.

Author	Title	Localization of EP	Patients	Treatment
Tong et al. (2016) [11]	Hepatoid Adenocarcinoma Arising from Heterotopic Pancreas of the ileum: A Case Report	Ileum	1	Partial resection of ileum

Kim and Nam (2015) [12]	Heterotopic Pancreas Presented as Duodenal Tumor with Obstruction	Duodenum	1	Removing the mass via duodenotomy

Sundaram et al. (2015) [13]	Isolated Ileal Pancreatic Heterotopia Causing Intussusception with Gangrene	Ileum	1	Partial resection of ileum

Kilius et al. (2015) [14]	Asymptomatic Heterotopic Pancreas in Meckel's Diverticulum: A Case Report and Review of the Literature	Meckel's diverticulum	1	Resection of Meckel's diverticulum

Andersen et al. (2015) [15]	Heterotopic Pancreas Is a Rare Cause of Bleeding and Intestinal Intussusception	Ileum	1	Partial resection of ileum

Monier et al. (2014) [16]	Heterotopic Pancreas: A Rare Cause of Ileo-Ileal Intussusception	Ileum	1	Partial resection of ileum

Okamoto et al. (2014) [17]	Intraductal Papillary Mucinous Neoplasm Originating from a Jejunal Heterotopic Pancreas: Report of a Case	Jejunum	1	Partial resection of jejunum

Wu et al. (2013) [18]	Adult Intussusception and Gastrointestinal Bleeding due to an Isolated Heterotopic Pancreas	Ileum	1	Partial resection of ileum

Ratan et al. (2012) [19]	Heterotopic Pancreas Leading to Ileo-Ileal Intussusception	Ileum	1	Partial resection of ileum

Lee et al. (2012) [20]	Ectopic Pancreas Bleeding in the Jejunum Revealed by Capsule Endoscopy	Jejunum	1	Partial resection of jejunum

Trifan et al. (2012) [21]	Gastric Heterotopic Pancreas: An Unusual Case and Review of the Literature	Stomach	1	Distal gastrectomy

Singh et al. (2012) [5]	Heterotopic Pancreas Presenting as Ileoileal Intussusception	Ileum	1	Partial resection of ileum

Yang et al. (2011) [22]	Massive Gastrointestinal Bleeding from Meckel Diverticulum with Ectopic Pancreatic Tissue	Meckel's diverticulum	1	Resection of Meckel's diverticulum

Seifarth et al. (2011) [3]	Diagnosis and Laparoscopic Treatment of Ileoileal Intussusception Secondary to Heterotopic Pancreas in an Infant: Case Report and Review of the Literature	Ileum	1	Partial resection of ileum

Bromberg et al. (2010) [2]	Pancreatic Heterotopias: Clinicopathological Analysis of 18 Patients	Stomach (7), duodenum (6), jejunum (3), gallbladder (1), and Meckel's diverticulum (1)	18	Distal gastrectomy (2), endoscopic resection (11), partial resection of jejunum (3), resection of Meckel's diverticulum (1), and cholecystectomy (1)

Gunjača et al. (2010) [23]	Inflammation of Ectopic Pancreatic Tissue as Unusual Cause of Duodenal Perforation: A Case Report	Duodenum	1	Distal gastrectomy with duodenum resection

Kopáčová et al. (2010) [24]	Inverted Meckel's Diverticulum with Ectopic Pancreatic Tissue as a Source of Severe Gastrointestinal Bleeding	Meckel's diverticulum	1	Resection of Meckel's diverticulum

Hirasaki et al. (2009) [4]	Jejunal Small Ectopic Pancreas Developing Jejunojejunal Intussusception: A Rare Cause of Ileus	Jejunum	1	Partial resection of jejunum

Seneviratne et al. (2009) [25]	Heterotopic Pancreas in the Body of the Stomach	Stomach	1	Total gastrectomy

Saka et al. (2009) [26]	Ectopic Pancreas as a Cause of Jejunal Obstruction in a Neonate	Jejunum	1	Partial resection of jejunum

Rana et al. (2009) [27]	Heterotopic Pancreas in the Jejunum Presenting as a Submucosal Lesion on Endoscopy	Jejunum	1	Partial resection of jejunum

Xiao et al. (2009) [28]	Heterotopic Pancreas within Meckel's Diverticulum with Obscure then Massive Gastrointestinal Bleeding in a 12-Year-Old Child: Case Report and Review of the Literature	Meckel's diverticulum	1	Resection of Meckel's diverticulum

Sautot-Vial and Steyaert (2009) [29]	Triple Intussusception Involving Heterotopic Pancreatic Tissue: A Case Report	Ileum	1	Partial resection of ileum

Gupta et al. (2014) [30]	Heterotopic Pancreas in Children: Review of the Literature and Report of 12 Cases	Meckel's diverticulum, (4), stomach (3), duodenum (3), jejunum (3), and ileum (2)	12	Endoscopic resection (6), partial resection of jejunum (3), resection of Meckel's diverticulum (4), and partial resection of ileum (2)

Kok et al. (2007) [9]	Adult Intussusception Caused by Heterotopic Pancreas	Jejunum	1	Partial resection of jejunum

## Abbreviations

EP: Ectopic pancreas.
